# Brain Vital Signs Detect Information Processing Differences When Neuromodulation Is Used During Cognitive Skills Training

**DOI:** 10.3389/fnhum.2020.00358

**Published:** 2020-09-14

**Authors:** Christopher J. Smith, Ashley Livingstone, Shaun D. Fickling, Pamela Tannouri, Natasha K.J. Campbell, Bimal Lakhani, Yuri Danilov, Jonathan M. Sackier, Ryan C.N. D’Arcy

**Affiliations:** ^1^Centre for Neurology Studies, HealthTech Connex, Metro-Vancouver, BC, Canada; ^2^Pavlov Institute of Physiology, Russian Academy of Science, Saint Petersburg, Russia; ^3^Helius Medical Technologies, Newtown, PA, United States; ^4^Nuffield Department of Surgical Sciences, Oxford University, Oxford, United Kingdom

**Keywords:** brain vital signs, electroencephalography, event-related potential, neuromodulation, neuroplasticity, PoNS^®^, cognitive training

## Abstract

**Background**: Neuromodulation through translingual neurostimulation (TLNS) has been shown to initiate long-lasting processes of neuronal reorganization with a variety of outcomes (i.e., neuroplasticity). Non-invasive TLNS is increasingly accessible through the Portable Neuromodulation Stimulator (PoNS^®^), a medical device that delivers electrical stimulation to the tongue to activate the trigeminal (V) and facial (VII) cranial nerves. Anecdotal reports from previous clinical studies have suggested incidental improvements in cognitive function. To objectively explore this observation, we examined TLNS-related effects on the semantic N400 brain vital sign cognitive response during cognitive skills training in healthy individuals.

**Methods**: Thirty-seven healthy volunteers were randomized to receive simultaneous TLNS (treatment) or no TLNS (control) while undergoing cognitive skills training. Cognitive training was conducted for two 20-min sessions (morning and afternoon/evening) over 3 consecutive days. Brain vital signs were evaluated at baseline, Day 1, and Day 3. Analyses focused on cognitive processing as measured by N400 changes in amplitude and latency.

**Results**: Over the 3-day course of cognitive training, the N400 amplitude decreased significantly in the control group due to habituation (*p* = 0.028). In contrast, there was no significant change in the TLNS treatment group.

**Conclusion**: TLNS led to a sustained N400 response during cognitive skills training, as measured by the brain’s vital signs framework. The study findings suggest differential learning effects due to neuromodulation, consistent with increased attention and cognitive vigilance.

## Introduction

### Neuromodulation and Neuroplasticity

A growing body of evidence suggests that translingual neurostimulation (TLNS) plays a role in modulating neuroplastic changes in the brain (Danilov and Paltin, 2018). The Portable Neuromodulation Stimulator (PoNS^®^), a Health Canada Class II approved medical device that applies sequenced, non-invasive stimulation to the tongue (Helius Medical Technologies, Newtown, PA, USA), is one such TLNS device. When placed on the tongue, the PoNS^®^ delivers electrical stimulation to the trigeminal and facial cranial nerves (CNV and CN-VII, respectively), which has been shown to modulate sensorimotor and vestibular functions (Herrick and Keifer, 1998; Buisseret-Delmas et al., 1999; Marano et al., 2005; Wildenberg et al., 2013). The stimulation is hypothesized to converge on and modulate visual, vestibular, nociceptive, and visceral sensory signals through bottom-up cerebellar and brainstem pathways to produce neuromodulation effects, affecting global brain function and augmenting neuroplasticity (Wildenberg et al., 2010; Frehlick et al., 2019). Prolonged stimulation has generated a variety of positive results, including the correction of gait and balance impairments when combined with physical therapy in the rehabilitation of individuals with brain injury (Leonard et al., 2017; Bastani et al., 2018; Danilov and Paltin, 2018; Tyler et al., 2019; Ptito, 2020).

### TLNS and Cognition

In early trials conducted using the TLNS device, participants with mild-to-moderate traumatic brain injury (mmTBI) anecdotally reported that, alongside the positive effects on movement and gait, they experienced improvements in their overall cognitive abilities (Danilov et al., 2015). Based on these incidental reports, it was hypothesized that adaptive changes occurring on different levels of brain organization (molecular, cellular, regional, and systemic) may extend beyond sensory and motor functions to cognitive performance and behavior (Danilov and Paltin, 2018). Accordingly, we conducted high-density electroencephalography (EEG) study investigating TLNS stimulation on health individuals using high- and low- frequency stimulation levels in a cross-over design (Frehlick et al., 2019). The results demonstrated significant changes in alpha and theta frequencies and, specifically, significantly increased activation of attention microstate activity. To date, however, direct evaluation of TLNS effects on cognitive function have not been investigated.

### Brain Vital Signs Framework

Evaluation of evoked brain responses through quantified EEG is increasingly applied as objective, physiological measurements of cognitive function (Gawryluk and D’Arcy, 2010). EEG-derived event-related potentials (ERPs; Luck, 2014), which represent brain responses to specific stimulus events, have been widely studied for this purpose. However, conventional ERP methodology and subsequent analyses are typically complex and time-consuming. To translate this laboratory capability into clinical applications, our group developed and validated a rapid evaluation platform known as the “brain vital signs” framework (Ghosh et al., 2016, 2018; Fickling et al., 2019). The brain vital signs framework employs a portable, low-density EEG system with automated, user-friendly software that facilitates the quick assessment of several key ERPs serving as brain function indicators.

Briefly, the brain vital signs framework extracts three well-established sensation-to-cognition target ERP responses (the N100, the P300, and the N400) that are elicited from a rapid 5-min auditory stimulation sequence comprised of randomly distributed auditory tones and spoken word pairs (Ghosh et al., 2016). Each ERP response is measured in latency (milliseconds) and amplitude (microvolts). The task delivered to the participants was a passive auditory task. Specifically, the stimulus was made up of 60 blocks of five tones followed by a primed word pair. Within each block of tones, one oddball deviant is randomly distributed to one of the five-tone positions. Out of the 60 sets of primed word pairs, half are congruent and half are incongruent. Of particular interest to the current study, the N400 indexes high-level cognitive processes during semantic processing (Kutas and Hillyard, 1980; D’Arcy et al., 2004, 2005; Kutas and Federmeier, 2010; Ghosh et al., 2018). Together, the N100, P300, and N400 measurements of brain function provide enhanced sensitivity to track cognitive changes in the brain. The brain’s vital signs framework has been successfully shown to be sensitive to cognitive changes in both healthy aging and brain injury (Ghosh et al., 2016; Fickling et al., 2019).

### Objectives and Hypothesis

We investigated whether TLNS affected cognitive processing in healthy individuals. To date, TLNS has been paired with physical therapy to enhance improvements in gait and balance (Leonard et al., 2017; Tyler et al., 2019). Accordingly, the current study investigated whether TLNS paired with cognitive skills training would significantly impact cognitive processing, as measured by brain vital signs in healthy individuals. The analysis concentrated on N400 response amplitudes and latencies as an indicator of high-level cognitive processing (Blackwood and Muir, 1990; Gawryluk and D’Arcy, 2010; Ghosh et al., 2016). We hypothesized that TLNS paired with cognitive training over 3 days would elicit N400 changes compared with cognitive training alone.

## Method

### Study Design and Conduct

This was a prospectively designed, single-center, randomized, controlled study conducted following the ethical principles that have their origin in the 1975 Declaration of Helsinki. The protocol was approved by a central institutional review board (Advarra IRB; Columbia, MD, USA).

### Participants

Thirty-seven (*N* = 37) healthy adult male and female volunteers were recruited for the study. Written informed consent was obtained from all participants before the administration of any study-related procedures. Participants were required to have a normal or corrected-to-normal vision, normal hearing, and self-reported healthy neurological function. Participants self-reported no concerns with healthy brain function and no further information was collected (e.g., prescription/recreational drug use). Statistical power analysis determined sample size estimation of between 30 and 50 volunteers. Participants were randomly assigned to treatment and control groups. Of the 37 volunteers recruited, eight participants were excluded from data analysis because they did not maintain either the cognitive training schedule or the EEG scanning schedule. Of the 29 participants included: 14 were in the Treatment Group (7M/7F, mean age = 29 ± 9 years old); and 15 were included in the Control Group (7M/8F, mean age = 30 ± 9 years old). Two additional participants were removed from the Control Group due to poor EEG signal quality.

### Study Procedures

**Figure 1:**
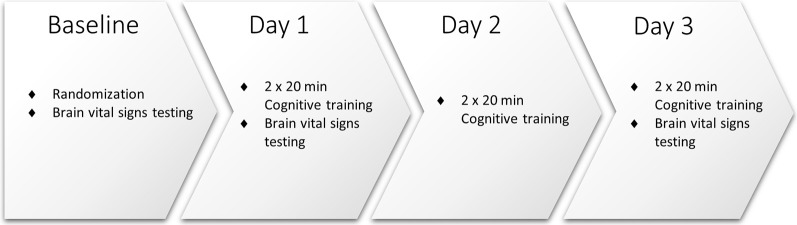
Study procedures.

As illustrated in Figure 1, study participants attended the clinic for baseline assessment and randomization, and on Days 1, 2, and 3. All participants underwent baseline brain vital signs testing on Day 1, following previously reported methods and outlined below (Ghosh et al., 2016, 2018). Pre- and post- neuropsychological testing was also conducted using the Single Digit Memory Test (SDMT) and the Paced Auditory Serial Addition Test (PASAT) as measures of information processing and working memory. Participants were then randomly assigned 1:1 to receive simultaneous TLNS (treatment) or no TLNS (control) while undergoing cognitive skills training.

**Figure 2:**
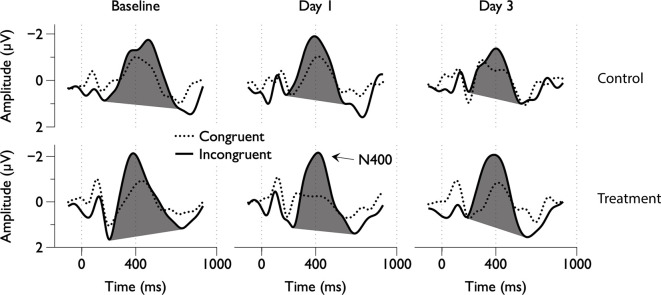
Group average electroencephalography (EEG) responses to congruent and incongruent word pairs across scan days.

All participants engaged in cognitive training twice a day (20 min, morning, and afternoon/evening) for three consecutive days. Cognitive training was performed using the Elevate™ brain training application on an iPad^®^ with a 9.7” light-emitting diode display (Apple, Cupertino, CA, USA). Participants were instructed to play the training regimen prescribed by the Elevate™, which utilizes all four categories available (i.e., writing, speaking, reading, and math). Once the prescribed training regimen was completed (approximately 10 min) participants were instructed to train further on any weak categories for the remainder of the training time. Participants randomized to the treatment group also received 20 min of simultaneous TLNS, administered *via* the investigational TLNS device. Brain vital signs were recorded once at Baseline, Day 1, and Day 3 (Figure 2). The PASAT and SDMT neuropsychological assessments were carried out again after study completion to evaluate any behavioral changes that may have resulted from the cognitive training.

### TLNS Treatment

The PoNS^®^ (Helius Medical Technologies, Newtown, PA, USA) device was used to deliver the TLNS stimulation. The device delivers 19-volt amplitude-controlled, pulse-width modulated, unbalanced biphasic pulses to the surface of the tongue. The stimulus is provided through 143 gold plated electrodes on a polyimide substrate. All participants in the treatment group received high-frequency pulse stimulation which consisted of triplets of 0.4–0.6 μs wide pulses at 5 ms intervals (i.e., 200 Hz) every 20 ms (50 Hz; Danilov et al., 2015; Danilov and Paltin, 2018; Ptito, 2020).

### EEG Data Collection and ERP Processing

As outlined in the brain vital sign framework (Ghosh et al., 2016), EEG was acquired using an 8-channel g.Nautilus EEG system (g.tec medical engineering, Austria) and three different 5-min auditory stimulus sequences randomly delivered from an HP Elitebook 840 G3 through Etymotic Research ER4 microPro earphones. All sequences used the same word pairs, however, the order of the word pairs varied. EEG data were recorded at 500 Hz, filtered using the onboard band-pass filter from 0.1 to 100 Hz along with a 60 Hz notch filter.

EEG data were processed to generate the ERPs for each participant. EEG data were bandpass filtered (0.5–20 Hz), baseline corrected, and ocular artifacts were removed through adaptive filtering using electrooculography (EOG) reference inputs. EOG was submitted to finite impulse response filters, followed by recursive least squares based artifact removal from the EEG signal (He et al., 2004). The EEG signal was then segmented and stimulus-averaged. ERP response latencies and amplitudes were recorded for the N400. Response amplitude was defined as the maximal peak amplitude with a pre-defined window for each ERP component and the response latency was calculated based on the time between the maximal peak and the stimulus onset.

### Statistical Analysis

Statistical analyses were performed in RStudio (Version 1.1463, RStudio Inc., Boston, MA, USA) using version 3.5.3 of the R statistical programming language (The R Foundation, Vienna, Austria). Mixed-effects linear regression modeled the amplitude and latency changes in each group using the lme4 package (Bates et al., 2015). Group (Control and Treatment) and Day (Baseline, Day 1, and Day 3) were analyzed as fixed effects with participants as the only random effect. Furthermore, a fixed interaction term of Group and Day was included. A likelihood ratio test was used to determine the impact of the fixed interaction term and the Group fixed effect. *Post hoc* Tukey adjusted pairwise contrasts (corrected for multiple comparisons) estimated marginal means using the emmeans package (Lenth, 2019). Both the SDMT and PASAT data were also analyzed with mixed-effects linear regression using the same fixed and random effects of the amplitude and latency models and were evaluated using likelihood ratio tests.

## Results

### Brain Vital Sign’s Data

**Figure 3:**
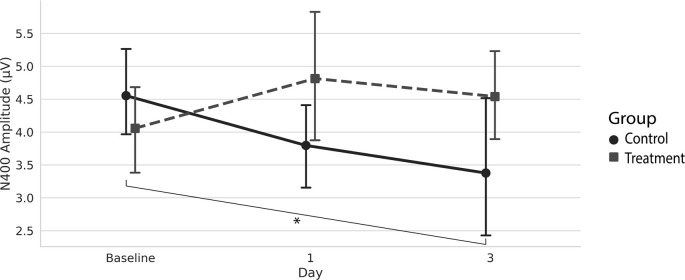
Average (with 95% confidence interval) N400 amplitude.*Denotes the significant interaction effect of Group and Day in the control group (*p* = 0.028).

Grand average ERPs were examined for Treatment and Control Groups across Baseline, Day 1, and Day 3, which confirmed the presence of the N100, P300, and N400. N400 response amplitudes differed between Groups as a function of Day (Figures 2, 3). In contrast to the N400 data, no notable results were detected for either the N100 or P300 components. Analysis of N400 ERP response amplitudes and latencies showed no significant difference between treatment and control groups at baseline (Table 1). However, there was a significant interaction effect of Group and Day for the N400 amplitude (χ^2^ = 7.1745, *p* = 0.028). The significant interaction resulted from the Control N400 amplitude reduction over the 3 days (Figure 3). In contrast, there was no significant change in Treatment N400 amplitudes over the 3 days.

**Table 1:** Brain vital signs N400 ERP amplitudes and latencies—means and standard deviations.

N400	Control (*n* = 15)	Treatment (*n* = 14)
Amplitude (μv)*
Baseline	4.55 ± 1.16	4.06 ± 1.37
Day 1	3.80 ± 1.17	4.81 ± 1.98
Day 3	3.38 ± 1.89	4.54 ± 1.43
**Latency (ms)**
Baseline	446.9 ± 108.2	401.1 ± 78.0
Day 1	376.6 ± 56.8	396.9 ± 77.5
Day 3	402.7 ± 68.8	397.7 ± 59.9

*p < 0.05, significant interaction. ERP, event-related potential. Post-baseline brain vital sign ERPs on each of Days 1 and 3 were measured following completion of cognitive skills training, which was paired with translingual neurostimulation (TLNS; Treatment) or without (Control).

### Cognitive Tests

Across participant groups, there was no statistical difference in behavioral performance on either the PASAT or SDMT.

## Discussion

Our findings demonstrate that pairing TLNS with cognitive training impacted the N400 markers of cognitive function over 3 days. Specifically, the N400 amplitudes remained stable for the Treatment Group across the 3 days, whereas the Control Group showed a significant amplitude reduction.

In the Control Group, habituation of N400 amplitude during repeat testing was expected. Within the brain vital signs framework, the cognitive N400 response is elicited from randomly distributed spoken word pair primes that are either semantically related/congruent (e.g., doctor-nurse) or unrelated/incongruent (e.g., doctor-egg; Ghosh et al., 2016, 2018). N400 habituation effects are well established in the literature, with the N400 to incongruent word prime pairs typically decreasing in amplitude with repeated stimulus exposure (Kutas and Federmeier, 2000). In the Treatment Group, however, the lack of habituation for N400 amplitudes represents, to our knowledge, a novel result.

Sustained N400 amplitudes, as seen in the Treatment Group, implicate underlying cognitive processes. In other words, sustained N400 amplitudes suggest sustained attention to word pair congruency during semantic processing (Federmeier and Kutas, 1999; D’Arcy et al., 2004). Interestingly, our prior high-density EEG study on TLNS effects in healthy controls also reported significantly increased spatial microstates associated with attention (Frehlick et al., 2019). Accordingly, the current findings support the role of TLNS in sustained attention (i.e., cognitive vigilance). Cognitive vigilance is the maintenance of an effortful process, such as semantic integration, to facilitate continued semantic integration during information processing. Thus, a sustained N400 amplitude suggests improved overall mental engagement with a task or learning exercise through enhanced cognitive vigilance. Future studies may wish to explore the role that TLNS plays in promoting sustained attention over longer periods.

Trigeminal and facial cranial nerve stimulation is hypothesized to induce a neuromodulator effect across multiple networks *via* the brainstem and cerebellum (Danilov and Paltin, 2018). In turn, this stimulation may facilitate neural activation across multiple systems involved in cognitive processing. However, specific mechanisms that underlie the effects of TLNS remain to be determined. Evidence from functional magnetic resonance imaging (fMRI) has shown that TLNS led to significant activation increases in the dorsolateral prefrontal and anterior cingulate regions (Klingberg, 2010; Leonard et al., 2017). These fMRI results suggest that TLNS may induce changes in regions associated with attention and working memory performance. Neural network models propose that interaction with attention processing directly influences working memory capacity (Klingberg, 2010; Eriksson et al., 2015), similar to attention influences on semantic processing in the current study. Accordingly, TLNS evidence in the literature across EEG, ERPs, and fMRI, suggest a model in which underlying attention activation facilitates improvements across interdependent cognitive abilities. Testing such a model represents an important next step to understanding TLNS effects on cognitive processing.

While the present study showed differences between Groups across the 3 days, there were notable caveats. Previous studies in which participants reported the cognitive and mental effects were significantly longer than the 3 days of this study. The short time frame used in this investigation may not characterize the full pattern of effects. Furthermore, previous studies investigating TLNS also involved pairing physical therapy and activity with stimulation, which was not included in the present study. Given that physical activity has long been reported to play a role in mental health and cognitive performance (Georgia et al., 2006; Kramer et al., 2006), this variable may have contributed to the mental and cognitive improvements reported in previous investigations.

This was an unblinded study conducted in healthy volunteers. It is challenging to include a sham comparison for TLNS studies as even low stimulations are not benign. Previous studies that have explored neuromodulation (Desantana et al., 2009; Silberstein et al., 2016), including other clinical research using TLNS (Ptito, 2020), have used sham devices with a minimally perceived low-frequency pulse. Results have shown significant effects from high or low-frequency pulse stimulation. Additionally, as previously noted in Frehlick et al. (2019), both high and low-frequency TLNS in healthy individuals affected alpha, theta, and attention-related activity to varying degrees. Thus, a valid sham for TLNS remains to be explored.

Additional research is required to better characterize underlying factors in TLNS across different clinical applications. Despite these caveats, the current results provide objective physiological evidence that TLNS paired with cognitive training led to subtle but significant differences in cognitive vigilance during semantic processing, suggesting that further investigation is warranted.

## Conclusions

The present study employed a brain vital signs framework to measure the effects of TLNS on cognition in a population of healthy individuals. We hypothesized that pairing TLNS with cognitive training (i.e., the Treatment Group) would elicit changes in cognitive processing, as measured by the N400 when compared with cognitive training alone (i.e., the Control Group). The Treatment Group showed a stable N400 response over the study duration, while the Control Group showed a significant decrease in response size; in other words, the Treatment Group demonstrated markers of increased sustained attention and improved cognitive vigilance.

## Data Availability Statement

The datasets presented in this article are not readily available because the datasets generated and/or analyzed during the current study are not currently publicly available due to intellectual property considerations. Requests to access the datasets should be directed to ryan@healthtechconnex.com.

## Ethics Statement

The studies involving human participants were reviewed and approved by Advarra IRB. The patients/participants provided their written informed consent to participate in this study.

## Author Contributions

CS collected, analyzed, and interpreted the ERP data and was the primary contributor to the manuscript. AL, SF, BL, PT, and RD’A all were involved in data collection, interpretation, and manuscript preparation. NC was involved in data interpretation and was a major contributor to the manuscript. YD and JS were involved in the study design and provided the PoNS**^®^** device for the study.

## Conflict of Interest

All authors report that they have financial and/or business interests in Helius Medical Technologies and HealthTech Connex Inc.
